# Communication Strategies Within the Mental Health Context—The Evidence for Nurses, Midwives, and Social Workers. A Rapid Review

**DOI:** 10.1111/inm.70371

**Published:** 2026-10-14

**Authors:** John Drayton, Glenda Hawley, Yee Kay Lai, Jacqueline Jauncey‐Cooke

**Affiliations:** ^1^ School of Nursing, Midwifery and Social Work The University of Queensland St Lucia Australia

**Keywords:** communication, mental health, midwife, nurse, social work, student

## Abstract

Effective communication in mental health settings is central to compassionate, ethical, and person‐centred care. For nurses, midwives, and social workers, communication underpins trust, assessment accuracy, and recovery‐oriented practice. This rapid review explored evidence‐based communication strategies used by these disciplines within adult mental health contexts. Following PRISMA‐ScR and JBI methodology, 18 studies published between 2015 and 2025 were analysed. Study quality was assessed using the Mixed Methods Assessment Tool. Three overarching themes emerged: professional mechanisms (reflective management, intuition, pre‐planning, rapport building), professional behaviours (presence, authenticity, non‐verbal communication, humour), and professional responses (validation, individualised approaches, strengths‐based care). Across studies, effective communication consistently enhanced therapeutic engagement, reduced distress, and supported recovery. Educational interventions, such as e‐learning, improved empathic communication but required ongoing reinforcement. Findings underscore communication as a teachable, modifiable skill integral to safe, high‐quality mental health care. This review is essential reading for novice nurses, midwives, and social workers entering mental health settings, offering practical, evidence‐informed guidance to optimise therapeutic interactions and promote recovery‐focused practice.

## 1 Introduction

Effective communication in mental health settings is a cornerstone of compassionate, ethical, and person‐centred care. For nurses, midwives, and social workers, the ability to engage in clear, empathetic, and culturally sensitive dialogue is critical not only for accurate assessment and treatment but also for building trust, reducing stigma, and supporting recovery‐oriented practice. Given the emotional complexity and vulnerability often present in mental health contexts, professionals must navigate a wide range of communication challenges—such as addressing diverse needs, managing anxiety and distress, and fostering interprofessional collaboration.

Challenges in communication are central to many mental health illnesses, creating additional complexity for healthcare professionals working in this area. Communication can be particularly challenging with people who are acutely anxious, distressed, or thought disordered. Ethical considerations and cultural and language differences add to the myriad of factors that contribute to the challenges of providing quality, person‐centred care within a mental health context. Additionally, the range of environments within which professionals care for people with mental health diagnoses of varying acuity has an impact on communication. Settings include community, acute inpatient, perinatal care, long‐term residential, forensic, and correctional. Each of these environments influences processes, management strategies, and opportunities around communication that must be considered.

Key professional organisations use position statements to provide guidance to clinicians or offer an explanation, a justification, or a recommendation for processes or practice. The Nursing and Midwifery Board of Australia (NMBA), the International Council of Nurses, International Confederation of Midwives, and the Australian Association of Social Workers (AASW) all publish professional standards and ethical frameworks that guide the practice of nurses, midwives, and social workers. These standards or position statements include domains around communication (AASW 2023; NMBA 2018; ICM 2024; ICN 2020). Additionally, the Australian College of Mental Health Nurses has specialist practice standards to guide clinicians across all settings (Australian College of Mental Health Nurses 2013). These specialist practice standards feature communication principles across several standards, with each describing different perspectives whilst emphasising the importance of interpersonal communication across all practice settings. Additionally, the Australian Commission on Safety and Quality in Health Care has the National Communicating for Safety Standard that demands health service organisations have processes in place that support effective communication with patients, carers, and families (Australian Commission on Safety and Quality in Health Care 2021).

Within these standards guiding practice there are various models of communication used within the wider healthcare setting. Therapeutic communication is a specialised form of communication that is purposeful and goal directed (Moxham et al. 2017). It involves establishing positive rapports with patients or clients based on mutual respect and engagement in the person's issues and concerns (Xue and Heffernan 2021). Through therapeutic communication, the emotional, psychological and social position of the person can be established and treatment plans or management strategies facilitated. Mindful or active listening, displaying positive non‐verbal cues, reflecting and paraphrasing all contribute to developing therapeutic communication (Xue and Heffernan 2021; Sharkiya 2023). Encouraging questions and avoiding the use of jargon are also important. All these cues indicate the health professionals' willingness to collaborate with the person; thus, they are more likely to experience a positive episode of healthcare. It may also contribute to an elevation in the person's sense of wellbeing, health status and self‐esteem (Sharkiya 2023). There are several other models or communication frameworks that have been used within healthcare or within healthcare education such as the Transactional Model of Communication and Calgary‐Cambridge Model (Silverman et al. 2013).

Despite communication being one of the vital interpersonal skills health professionals should possess, various studies have illustrated that many health professionals are lacking the confidence in using therapeutic communication, especially nurses, midwives, and social workers within the mental healthcare settings. Derblom et al. (2022) found that nurses are not confident with caring for patients with a mental illness as they are unsure of what to say or do with these patients and fear they may aggravate the situation. Physical and verbal aggression remains a common critical incident within acute mental health facilities (Parakkal Kurian et al. 2023). Noonan et al. (2018) found that midwives possess high levels of confidence (72%) and knowledge (71%) in identifying women who experience depression and anxiety; however, only 17.8% (*n* = 28) felt confident in supporting women with perinatal mental health illness. Mental health settings often involve intricate communication scenarios, requiring advanced skills to navigate sensitive topics, manage crises, and engage with individuals experiencing severe mental distress. Clinicians may find these situations particularly challenging.

Rather than critique various models or frameworks, this review aims to explore the evidence for communication strategies with people within the mental health context, with a focus on nurses, midwives, and social workers. The term ‘mental health context’ refers to the environment, conditions, and systems in which mental health care is delivered, experienced, and understood. The aim of this review is to provide clinicians with clear, contemporary evidence on optimising communication within the mental health context. Additionally, in this review, the terms ‘person or people’ will be used primarily. This term is inclusive of client, woman, service user, or patient as per the authors of the referenced studies.

## 2 Methods

This rapid review is reported following the guidance provided by the Preferred Reporting Items for Systematic Reviews and Meta‐Analyses extension for Scoping Reviews (PRISMA‐ScR) Checklist and the Joanna Briggs Institute JBI scoping review methodology (Stevens et al. 2025; Hanan et al. 2016). The review was prospectively registered within the Open Science Framework 2025 (https://osf.io/yftd8/).

### 2.1 Study Selection and Eligibility Criteria

#### 2.1.1 Inclusion Criteria

Studies were considered for inclusion that explored communication strategies within the adult mental health context. Original qualitative or quantitative research that focused on therapeutic communication and de‐escalation techniques used by nurses, midwives, and social workers was included. All included studies were peer‐reviewed, written in English and published between January 2015 and January 2025.

#### 2.1.2 Exclusion Criteria

Studies that focused on children or adolescents were excluded. Studies that focused on communication within the context of neurodivergence or disabilities were excluded. Studies that focused on dementia including Alzheimer's were also excluded. Review methodologies were excluded, as were conference abstracts, editorials, and protocols.

#### 2.1.3 Search Strategy

Following consultation with a University Librarian, a comprehensive search strategy was developed. The Cumulative Index to Nursing and Allied Health Literature (CINAHL) Complete, PubMed, and Scopus databases were searched for articles on communication and mental health. Using a rapid review approach, grey literature was not included in the search. A Boolean strategy was applied with database‐specific variations on the search terms ‘mental health’ OR ‘mental health disorders’, OR ‘mental health illness’ AND communication OR conversation AND nurse (‘nurses’), midwife (‘midwifery’), social worker (‘social workers’). These terms were adapted and entered into CINAHL and Scopus. A file was created within Covidence systematic review software (Veritas Health Innovation, Melbourne, Australia. http://www.covidence.org) with the agreed inclusion and exclusion criteria. All authors were provided access to the Covidence file. The results from three databases were then uploaded into Covidence via Endnote 2025 (Clarivate 2025), and duplicates were recognised and automatically removed by the platform.

#### 2.1.4 Selection Process

After the search results were uploaded into Covidence, the titles and abstracts of the studies were independently reviewed by two authors based on the inclusion and exclusion criteria. Full text screening was then undertaken by two authors. Any discrepancies or disagreements were reviewed collectively and resolved by consensus at each stage of the screening process. Articles were included if they were published in English, peer‐reviewed, and focused on communication for healthcare professionals working in mental health care environments. All authors read all included studies.

#### 2.1.5 Data Extraction

During the data extraction process, the main characteristics and key findings of the included studies were recorded using a purpose‐built data extraction tool; these were then exported and presented in a table. The table was then reviewed by two authors to ensure all relevant information was presented and to minimise the risk of bias.

#### 2.1.6 Study Quality Appraisal

The quality of the included studies was appraised using the Mixed Methods Appraisal Tool (Hong, Fàbregues, et al. 2018). Whilst a quality assessment is not imperative within a rapid review methodology, the authors wanted a means by which to assess the strength of reporting within the included studies (Hong, Fàbregues, et al. 2018). To achieve consistency of assessment across the MMAT tool, one researcher assessed all studies (Y.K.L.), and two researchers each reviewed half of the total number of studies (G.H. and J.D.). Any discrepancies were resolved by discussion between the researchers.

The overall MMAT score was presented with an asterisk (*); the number of * represents the number of quality criteria the study has met, with a total of five asterisks equating to a value of 100%. The quality appraisal percentage for each included study is detailed in Table 1.

**TABLE 1:** Included studies.

Author, year, country	Study design	Study Aims	Setting	Key findings	MMAT score
Berg et al. (2023) Finland	Qualitative Focus group interview	Improving nurse‐patient communication during patient seclusion	Mental health inpatient	Clear and direct communication is vital, including giving straightforward answers and informing patients about treatments or assessments. Individualised care ensures that each patient feels seen and respected. Nurses enhance therapeutic communication by being present and attentive, noticing subtle gestures, and maintaining a calm, natural demeanour.	*****
Chambers et al. (2015) UK	Qualitative Content analysis	Establish the experience of nurses when caring for distressed and disturbed people	Acute mental health inpatient	Staff experience anxiety, fear and vulnerability. They sometimes avoid difficult conversations or situations, largely to be self‐protective. Ascertaining and managing risk are incredibly challenging. Cognitive dissonance around managing these situations within policy or guidelines which can't apply to every situation. Also the disconnect between training and applying the principles to real world events. Therapeutic engagement and building and sustaining a therapeutic relationship is highly valued. Listening is important; be mindful of tone of voice, posture and the content of your conversation; permit time to reflect when distressed and follow up later. Experienced colleagues, organisational support and ongoing education and training is essential.	*****
Delaney et al. (2017) USA	Qualitative Preliminary renderings of the model	Create a model that outlines the process of interpersonal engagement (6 steps)	Review	To foster meaningful interpersonal engagement, nurses should begin by centering themselves and clearing their minds. They must be fully present, actively listen without interrupting, and remain aware of their body language. Establishing an empathic bridge involves attuning to the patient's expressions and actions. Understanding the patient's story—through their lived experiences—enables nurses to craft intuitive, responsive communication tailored to each interaction.	**
Evans et al. (2017) USA	Mixed methods Nested study	Examine a nurse‐delivered telephone support intervention to low‐income pregnant women in rural settings	Telephone support	Telephone support intervention modelled around Peplau's theory of interpersonal relations; engage, listen, interact, outcomes. Nurse delivered telephone support intervention may be an effective way of providing support women in rural areas and may offset antepartum depression.	***
Finley et al. (2024) USA	Phenomenology	Explore psychiatric mental health nurse practitioners' experiences with therapeutic alliances using tele‐mental health	Telehealth	Nurse practitioners support patients through attentive listening, clarifying questions, and reflective, interpersonal techniques. Psychotherapy is tailored to individual needs, sometimes requiring gentle redirection. Tele‐mental health enhances care by revealing home conditions like hoarding and promoting family involvement, offering deeper insight and broader support for the patient's therapeutic journey.	*****
Griep et al. (2016) Netherlands	Cross‐sectional observation Analyse recorded video	Examine how patients with mild psychosocial and or psychological ‘complaints’ were expressed and examine how nurses responded	CommunityGeneral practice	Practice nurses mental health working with community general practice settings participated in the study. In almost all consultations people expressed ‘complaints’. The participants scored highly in: having passive listening skills and providing space for patients to talk about their cue or concern. There was no single approach that is considered best to respond and people value responses differently.	****
Korteland et al. (2019) Netherlands	Qualitative	Identify nursing interventions used at a mental health mother‐baby unit, when the woman has postpartum psychosis	Mental health mother‐baby unit	During the acute phase of admission for people with post partum psychosis participants identified that their focus is initially on assessing, stabilising, and providing information, while also addressing breastfeeding possibilities, attending to self‐care, and ensuring infant safety. Maximising mother‐baby interaction is prioritised throughout care. Treatment involves educating and supporting parenting, and during discharge, participants aim to support people to balance activity and rest. Structured psychoeducation is provided to prevent relapse, supported by a consistent daily schedule.	*****
Lim et al. (2021) Australia	Q‐methodology	Understand how nurses use recovery‐focused care in the acute mental health settings to reduce the risk of aggression	Acute mental health settings	Recovery‐focused care prioritises empathy, reassurance, and trust‐building through meaningful nurse–patient interactions. By fostering safe spaces, avoiding restrictive practices, and encouraging self‐regulation, patients regain control and confidence. Trauma assessment guides tailored interventions, while reflection deepens understanding. Ultimately, recovery‐focused care empowers individuals to own their healing journey, reducing aggression and promoting lasting change.	****
Lin et al. (2022) Taiwan	Qualitative Content analysis	Study aimed to explore strategies employed by nurses to promote medication adherence in patients with schizophrenia	Inpatient mental health facility	Establishing a conversational relationship and building trust is key as is showing empathy, listening, and validating patient concerns. Understanding medication refusal through gentle questions and relatable analogies encourages openness. Explaining benefits, offering affirmations, and sharing anecdotes support adherence. Emphasising reduced caregiver burden and promoting shared decision‐making empowers patients to engage actively in their recovery journey.	****
Molin and Graneheim (2022) Sweden	Qualitative Content analysis	Explore the nursing staff's experience of the nursing intervention ‘Daily Conversations’	Inpatient mental health facility	Daily Conversations is a structured nursing intervention that ensures that patients' need for conversation were met. Through the process of Daily Conversations nurses were able to connect with patients, fostering trust, visibility, and person‐centred care. Though some patients find them artificial, open, flexible dialogue reveals health changes and reduced medication use. Nurses required training and management support, as documentation burdens and lack of confidence can hinder meaningful engagement, especially with complex mental health cases.	*****
Oestergaard and Dinesen (2019) Denmark	Qualitative Case study method	Explore how social workers experience video communication as part of a tele‐social‐rehabilitation program.	Telehealth/video technology community setting	Participants found that video communication was an excellent alternative to home visits. It was less intrusive than physical visits and offered a new way to collaborate with vulnerable clients. It promoted recovery by encouraging self‐reliance and openness in conversations, more so than traditional home visits. Additionally, video communication helped reduce clients' feelings of isolation and anxiety.	****
Overpelt et al. (2025) Netherlands	Qualitative	Study aims to learn how nurses foster therapeutic relationships with clients who display conflict behaviour	Acute mental health setting	The three main themes discovered were; co‐operation, structure and safety. Nurses foster therapeutic relationships with clients displaying conflict behaviour by modelling healthy actions, maintaining clarity, exploring options collaboratively, and encouraging trial‐and‐error practice of productive behaviours.	*****
Scheirich et al. (2024) Australia	Mixed methods Experimental design	Study aimed to develop and test a recovery‐oriented script to guide communication with people at imminent risk of aggression	Secure Forensic Mental Health Hospital	Co‐designed scripts developed to encompass recovery‐oriented principles and best practice. Scripts underwent testing by experts in forensic mental health nursing and lived experience expertise. Both scripts could prevent aggression because they acknowledge the consumer's experience and demonstrated understanding of the consumer's perspective. Scripts encourage consumers to participate in decision‐making process. Scripts anticipated to reduce the risk of aggression within the forensic setting.	****
Shinohara et al. (2022) Japan	Randomised controlled trial.	To evaluate the effects of e‐learning education for empathic communication skills and perinatal mental health knowledge	Educational setting	A 1‐day educational intervention for midwives, nurses and public health nurses employed within a perinatal setting. The intervention generally improved knowledge in conducting perinatal mental health assessments and increased rates of empathic communication.	****
Spielfogel and McMillen (2017) USA	Group concept mapping	Identify practices used to de‐escalate behaviour in people in distress	Online study	Participants included any professional that works with people that potentially exhibit signs of distress such as nurses, social workers, education, law enforcement, mental health. Through several phases of sorting and analysis the findings included the following; validation, listening and body language are rated the most important in terms of de‐escalation. There was also an emphasis on environmental safety. The Mental Health group rated additional support and problem solving as more helpful. Mental Health group rated additional support and problem solving as more helpful.	*****
Tolosa‐Merlos et al. (2023) Spain	Participatory action research	Explore the concept of therapeutic relationship through the nurses' reflective practice	Acute Mental Health Unit	From reflective diaries it is apparent that there are three core categories; nursing attitude is key to the therapeutic relationship, approach to practice is essential in building a therapeutic relationship and finally context influences practice. Nurses build therapeutic relationships by maintaining a positive attitude, being open and available, and offering time. They pay close attention to verbal and non‐verbal cues, use a calm tone, show interest, and maintain appropriate physical boundaries. Care is individualised, and the environment is structured to support therapeutic engagement, avoiding mechanical restraints.	*****
Unhjem et al. (2018) Norway	Qualitative	To describe what and why nurses self‐disclose in practice	Local mental health services—multisite	Nurses foster therapeutic relationships by self‐disclosing aspects of their personal lives—such as family, interests, and experiences—to patients in one‐on‐one interactions. This approach promotes openness, reciprocity, and equality in the nurse–patient relationship, helps normalise patients' experiences, models coping strategies, and supports a more natural and honest dialogue.	*****
Vandewalle et al. (2019) Belgium	Grounded theory	Understand how nurses in mental health inpatient settings connect with patients experiencing suicidal ideation	Inpatient mental health hospitals—multisite	Careful risk assessment by using a checking approach. Create an environment that supports open and genuine communication to get to know the patients and develop an understanding of the patient's suicidal ideation. Maintain a focus on establishing a relationship by initiating contact, being present, accessible and approachable. Active listening, direct questions and observe for non‐verbal cues. Some participants describe using creative methods (such as drawing) to support discussions around suicide ideation.	*****

*Note:* The authors of the MMAT tool prefer that stars are used to depict the results of the quality assessment rather than a number.

#### 2.1.7 Data Synthesis

Quantitative and qualitative studies were synthesised separately due to their differing methodological designs and outcomes of interest, and because the small number and heterogeneity of the quantitative studies (*n* = 3) precluded meaningful integration with the qualitative findings. Quantitative studies were synthesised to textualise the effectiveness of interventions or compare observational data (Campbell et al. 2020). Qualitative studies utilised descriptive content analysis guided by methods described by Saldaña (2025). Following content analysis, each study was labelled with a code. Multiple codes that share a similar meaning were then grouped together to identify a theme. This process was repeated multiple times to identify different themes presented across the included studies (Saldaña 2025).

## 3 Results

The Preferred Reporting Items for Systematic and Meta‐Analyses (PRISMA) statement was used to guide the execution and reporting of this review (presented as Figure 1).

**FIGURE 1:**
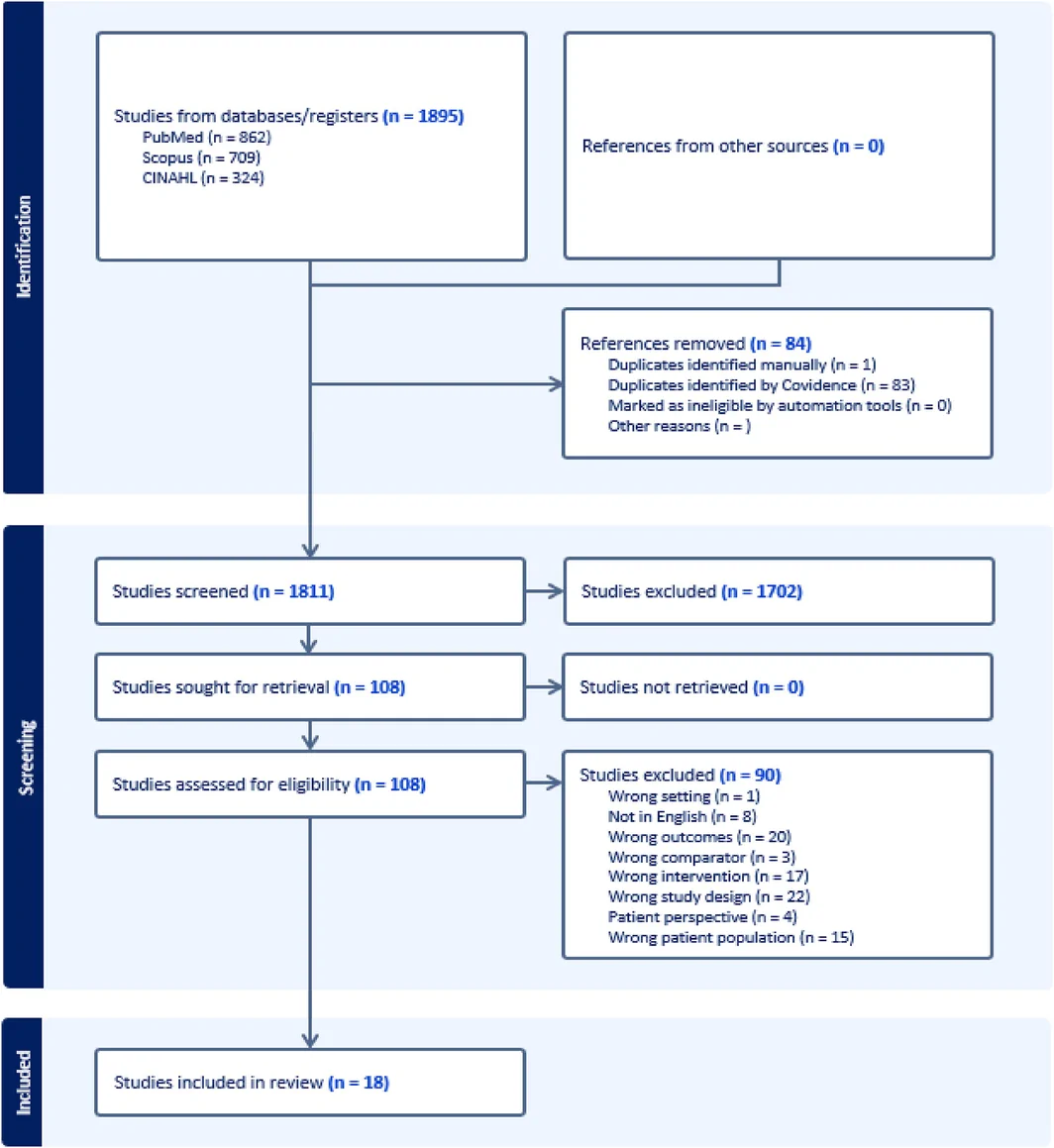
Prisma flow diagram.

The systematic search of literature was initially undertaken in February 2025. A total of 1895 records were identified, including 83 duplicates. The remaining 1811 were screened by the title and abstract (Figure 1). A total of 108 full text studies were assessed for eligibility against the inclusion criteria. Reasons for exclusion are noted in Figure 1.

A total of 18 studies were included in the final analysis. The key characteristics of studies included in the review are described in Table 1.

### 3.1 Description of Included Studies

Of the 18 studies included in the final analysis, 50% were based within an inpatient setting; two studies were based in community mental health settings, two investigating telehealth as a platform for communication and one publication that developed a conceptual model to support communication and interpersonal processes. Qualitative methodologies were used in the majority of studies (Berg et al. 2023; Chambers et al. 2015; Delaney et al. 2017; Korteland et al. 2019; Lin et al. 2022; Molin and Graneheim 2022; Oestergaard and Dinesen 2019; Overpelt et al. 2025; Scheirich et al. 2024; Spielfogel and McMillen 2017; Tolosa‐Merlos et al. 2023; Unhjem et al. 2018; Vandewalle et al. 2019). Quantitative methodologies were used in three studies (Griep et al. 2016; Lim et al. 2021; Shinohara et al. 2022) and one study (Scheirich et al. 2024) used a mixed methods approach.

The majority of included studies focused on the profession of nursing (78%), almost 20% were applicable to midwifery, one study focused on social work practitioners and one study using an interprofessional approach. Internationally, studies were undertaken in Australia (Lim et al. 2021; Scheirich et al. 2024), United States of America (Delaney et al. 2017; Evans et al. 2017; Finley et al. 2024; Spielfogel and McMillen 2017), Netherlands (Griep et al. 2016; Korteland et al. 2019; Overpelt et al. 2025), United Kingdom (Chambers et al. 2015), Finland (Berg et al. 2023), Taiwan (Lin et al. 2022), Sweden (Molin and Graneheim 2022), Denmark (Oestergaard and Dinesen 2019), Japan (Shinohara et al. 2022), Spain (Tolosa‐Merlos et al. 2023), Norway (Unhjem et al. 2018), and Belgium (Vandewalle et al. 2019).

### 3.2 Qualitative Study Synthesis

Qualitative findings were synthesised iteratively: data extracted from each study were read and re‐read to identify recurring concepts, which were recursively grouped into candidate themes and sub‐themes (Saldaña 2025). This process resulted in three overarching themes, each comprising four sub‐themes, with groupings refined through discussion among the review team until consensus was reached.

#### 3.2.1 Theme 1—Professional Mechanisms

The term professional mechanisms refers to the purposeful use of practice strategies to facilitate effective relational interactions with the patient. The term “mechanisms” has been used to emphasise the purposeful nature of the strategies.

##### 3.2.1.1 Reflective Management of Self and Responses

Self‐management, particularly in regard to challenging or confronting behaviours from patients, is recognised as a necessary skill in mental health work (Berg, Chambers, Lin). Studies engaging with this theme discuss ways of maintaining equilibrium and a sense of role recognised as a means of ensuring the professional's responses to what in another context may seem to be a provocation.

Some studies emphasise the utility of a “mindful” approach (Chambers) to vocal tone, posture and conversation content, or a process of self‐centring, in which the professional clears the mind prior to meeting with the patient (Delaney et al.). In both cases, the goal is establishment of a calm demeanour (Berg), modelling positive behaviours for the patient (Overpelt et al., Unhjem et al.). Tolosa‐Merlos et al. (2023) extend this to considering the environment in which the professional interacts with the patient. They note the importance of a calm, comfortable environment without external stimuli or interruptions in establishing and maintaining therapeutic relationships.

Korteland et al. (2019) and Lin et al. (2022), suggest the professional's self‐management should focus on the development of trust with the patient—an outcome they relate to an attitude of acceptance and the willingness to explain to patients why certain treatment decisions have been made. Both studies link the management of self and responses to improved health outcomes, be it medication compliance (Lin et al.) or engagement with proposed interventions (Kortelen et al.).

##### 3.2.1.2 Intuitive Approach

In contrast to the reflective, mindful approach, the literature reviewed also recognised the role of experience‐derived intuitive practice, not unrelated to the exercise of empathetic engagement.

Delaney et al. (2017) point to the importance of “attunement” in practice. By this, they refer to the process whereby nurses interpret the patient's actions and expression; intuiting what the patient is thinking and feeling while remaining conscious of their own thoughts. This phenomenologically complex process actually combines reflection and intuition: the nurse maintains awareness of their own position (a reflective stance) even while intuiting that of the patient. The reflective element here protects against projection.

Similarly, Vandewalle et al. (2019) discuss in the context of suicidality. Experience and familiarity with the patient, they suggest, enable the professional to intuit a sense of the suicidal patient's lack of hope, directly informing risk assessments.

Unhjem et al. (2018) discuss the issue of self‐disclosure from nurse to patient. This practice again appears to involve a complex linking of reflection and intuition. Nurses' decisions to self‐disclose are reported as arising from clearly considered positions such as building a therapeutic relationship and equalising power relations. At the same time, the decision to proceed with sharing is a largely intuitive one, based on a sense of the conversation feeling “natural” and avoidance of issues which feel in some way “awkward”.

##### 3.2.1.3 Pre‐Planning of Communication and Preparation

In direct contrast to intuition‐informed communication strategies, pre‐planning and preparation are noted as particularly significant in relation to working with patients in restraints. By their nature, restraints are considered unhelpful in maintaining a therapeutic relationship (Tolosa‐Merlos et al.). Berg et al. (2023) discuss focus group findings which suggest nurses themselves find it difficult to communicate optimally when a patient is being restricted and suggest the importance of planning in advance how any conversations should be managed. The issue of pre‐planning is contested in some cases. Molin and Graneheim (2022) note that the Daily Conversation method (in which the professional plans each day to interact with patients) is beneficial in enabling both the creation of mutual trust and for professionals to focus more on the patient's stories and engage in person‐centred care. Evans et al. (2017) affirm this concept of planned support with pregnant women by utilising telephone intervention, based on Peplau's theory of interpersonal relations. That includes an intentional approach to engaging, listening, interacting, and outcome focused (Evans et al. 2017). At the same time, however, patient feedback suggests that some consider the approach to be forced and uncomfortable. Some nurses, too, have found the pre‐planned aspect to introduce a degree of artificiality into their work and have noted that the pre‐planning element has resulted in the need for such exchanges to be documented, a further challenge to therapeutic informality.

##### 3.2.1.4 Rapport Building

The development of rapport lies at the heart of professional communication mechanisms, and all reviewed papers make at least an oblique reference to the practice.

Empathy is fundamental to the development of rapport. Delaney et al. (2017) refer to the need for practitioners to develop empathy using the ‘bottom up’ approach or the ‘top down’ approach. The former approach occurs when the nurse is aware of the patient's mood, while ‘Top Down’ approach occurs when the nurse can imagine what it is like to be in the person's shoes.

Other skills linked in the literature with rapport building include taking care to explain outcomes and treatment processes to patients (Berg et al.), asking clarifying questions (Finley et al.), validating patients' narratives of lived experience, and providing reassurance and dedicated time (Lim et al.).

Finley et al. (2024) note the importance of attentive listening to rapport. This general approach can be associated with attending to matters of non‐verbal cues, vocal tone, displays of interest and body language (Tolosa‐Melon et al.). Griep et al. (2016), in a study of nurse responses to patient complaints, note a range of pertinent skills, including acknowledgement, the use of silence, active invitation, affective exploration and empathy, and the provision of advice.

#### 3.2.2 Theme 2—Professional Behaviours

The term professional behaviours refers to the observable conduct and interpersonal qualities that clinicians display during interactions with people, distinct from the purposeful strategies captured under professional mechanisms. While professional mechanisms describe *how* clinicians plan and self‐regulate to support communication, professional behaviours describe *what* clinicians visibly do and convey in the moment of interaction—namely, presence and authenticity, non‐verbal communication, and the use of humour.

##### 3.2.2.1 Presence and Authenticity in All Interactions

A number of included studies emphasised the importance of clinicians being ‘present’ during episodes of communication (Berg et al. 2023; Delaney et al. 2017; Finley et al. 2024; Lin et al. 2022). For communication to be effective, the person must have a genuine sense that the clinician is engaged in the conversation, that they are focused on the person in the moment and actively listening (Berg et al. 2023; Delaney et al. 2017; Finley et al. 2024; and Lin et al. 2022). Authenticity and focus were considered to be imperative in developing a rapport with people and are demonstrated principally by eye contact and non‐verbal communications. Additionally, distractions need to be minimised and sufficient time given to the interaction.

##### 3.2.2.2 Non‐Verbal Communication

Insight and awareness of non‐verbal communication was also described across a number of the included studies. Of the 18 papers included in the final analysis, nine included the role of non‐verbal communication in establishing a therapeutic relationship (Berg et al. 2023; Chambers et al. 2015; Delaney et al. 2017; Korteland et al. 2019; Lin et al. 2022; Molin and Graneheim 2022; Overpelt et al. 2025; Spielfogel and McMillen 2017; and Tolosa‐Merlos et al. 2023). Each of these studies used qualitative methodologies to explore various aspects of communication, including the role of non‐verbal communication. Their findings emphasise the role of eye contact, posture, positioning, and expressing care non‐verbally, perhaps through therapeutic touch where appropriate. These studies collectively underscored that non‐verbal communication often conveys meaning more powerfully than spoken words, particularly in sensitive or emotionally charged interactions (Chambers et al. 2015; Molin and Graneheim 2022). Clinicians' ability to recognise and appropriately respond to subtle cues—such as a patient's facial expressions, tone of voice, or physical withdrawal—was seen as central to demonstrating empathy and validating the patient's experience. Conversely, incongruence between verbal and non‐verbal messages, such as a dismissive tone or lack of eye contact, could undermine trust and rapport. As such, developing self‐awareness and reflective practice around non‐verbal behaviours was viewed as a critical component of effective therapeutic communication.

##### 3.2.2.3 Humour

A range of communication strategies were discussed in the included studies, with humour being rated as an important element of a therapeutic relationship (Finley et al. 2024; Vandewalle et al. 2019). Humour may be an effective way to create connections with people and develop that essential rapport required in an open, reciprocal, therapeutic relationship. Humour is an important adjunct to communication and can also be used to promote a therapeutic alliance through telehealth. Importantly, the use of humour in clinical interactions was found to require sensitivity, timing, and awareness of individual preferences and cultural contexts (Finley et al. 2024; Vandewalle et al. 2019). Appropriate humour—particularly when initiated in response to patient cues—can humanise the clinician, reduce tension, and foster a sense of mutual respect and equality within the therapeutic relationship. However, studies cautioned that humour used without attunement to the patient's emotional state or circumstances may risk misunderstanding or offence. Consequently, clinicians are encouraged to use humour judiciously as a means of enhancing connection and trust, rather than as a distraction or avoidance of difficult emotions.

#### 3.2.3 Theme 3—Professional Responses

This theme identified techniques used by healthcare professionals in managing and communicating with people. Within this theme, 4 sub‐themes were identified. Of the 18 included papers, 6 contributed to this theme (Delaney et al. 2017; Lim et al. 2021; Scheirich et al. 2024; Korteland et al. 2019; Molin and Graneheim 2022; Lin et al. 2022).

##### 3.2.3.1 Validation of People's Experiences

Attunement is a cornerstone to the interpretation of a person's actions. This is a means to gain insight into their thoughts and emotions while maintaining awareness of your own internal responses (Delaney et al. 2017). The connection is therapeutic to support emotional safety and trust, which is considered an essential component in the care of individuals with a trauma‐involved history. Evans et al. (2017) applied a multi‐method, 4‐phase Peplau approach to build a relationship based on evidence and validation of psychological mental health scores of pregnant women at the commencement of the nurse‐client interaction. Once identified, the nurse was able to contextualise the trend of quantitative scores over the time of the pregnancy to verify the qualitative iterative approach used in a telephone intervention with women. This approach facilitated the layered coding steps in the analysis and informed conclusions (Evans et al. 2017).

Lim et al. (2021), identifies that a comprehensive assessment is necessary to determine initially the risk for potential aggression, in order to tailor interventions that are both preventative and responsive for that person. By encouraging people to reflect on the impact of their trauma on current behaviours, this will promote self‐awareness to foster an understanding of their own responses. Lim et al. (2021), highlights that using positive reinforcement will help people to regain control of their good behaviour.

Scheirich et al. (2024) describe empathy as central to validating people's experiences, particularly in regard to aggression. Nurses convey empathy by acknowledging and understanding a person's feelings and reasons for the aggressive behaviour. This is seen as a collaborative approach with the person with shared decision making and is often perceived as a form of respect. It also provides people with options to be involved in their own treatment.

##### 3.2.3.2 Individualised Approach

This sub‐theme aligns with connecting the physical wellness with the psychological. By promoting rest, restoring sleep patterns and providing a safe environment, a daily routine can form to build structure, trust and improved physical wellness (Korteland et al. 2019; Molin and Graneheim 2022). An individualised approach necessarily involves addressing the specific circumstances of the person and their diagnosis. In the context of postnatal psychiatric care, for example, Korteland et al. (2019) discusses the role of a nurse or midwife as central to assessing the level of supervision the mother requires when caring for her baby. The nurse or midwife should understand the previous history and observe the mother's current condition to ascertain the risk associated to the infant's safety if the diad is left alone. provides advice and emphasises the mother's responsibility for her baby (Korteland et al. 2019). By interacting with the woman closely, the nurse or midwife takes a supportive approach, to also check for any postnatal physical issues of blood loss, and possible breast engorgement or attachment problems when feeding (Korteland et al. 2019).

##### 3.2.3.3 Strengths Based

This sub‐theme demonstrates the importance of communication by emphasising each person has an important role to play in their own recovery. Nurses play an important role in encouraging people to be decision makers in their own treatment, including medication prescription by the doctor (Lin et al. 2022). This approach acknowledges the lived experience and validates the feelings of the person, thereby promoting trust with the health care team while helping them to develop coping strategies (Lin et al. 2022; Scheirich et al. 2024).

However, Scheirich and colleagues highlight that the success of this approach is dependant on the willingness of the person to cooperate and may be limited at times of acute mental distress (Scheirich et al. 2024).

### 3.3 Narrative Summary of Studies Using Quantitative Methodologies

Quantitative studies were narratively synthesised, following the Synthesis Without Meta‐analysis (SWiM) guideline (Campbell et al. 2020), to summarise intervention effectiveness and compare findings across observational designs. The Griep et al. (2016) study explored Dutch Mental Health Practice Nurses responses to patient's expressing concerns. This observational, exploratory study used the Verona Coding; VR‐CoDES‐CC (Zimmermann et al. 2011), a validated framework to code and quantify cues and concerns observed in video recorded consultations. Fifteen practitioners were observed over 117 consultations. In 109 of these consultations cues or concerns were expressed and the practitioners' responses were coded into four categories as follows; non‐explicit reducing space, non‐explicit providing space, Explicit reducing space and explicit providing space. The most common response was non‐explicit providing space and this was identified by either acknowledging, implicit empathy, silence, active invitation or what the authors refer to as back channelling. Back channelling describes any response that encourages further disclosure through using a minimal prompt, for example, mmm, yes, right or okay. Of all the observed cues and concerns only 14% of responses were coded as ‘reducing space’ for further disclosure which illustrates the expertise of the clinicians observed.

The study by Lim and colleagues used Q‐methodology to explore communication strategies that expressly supported recovery focused care (2021). Through purposive sampling, the authors sought experienced mental health nurses from across Australia to organise a series of statements across a continuum. Ultimately, five enabling factors were identified by participants: acknowledgment of the emic experience of the person, reassurance of difficulties, interaction to explore the person's lived experiences, collaboration to reduce triggers, and encouragement and support for people to assume ownership of their recovery journey. These factors all support a reduction in the potential for aggression and support recovery outcomes through therapeutic communication. This study concluded that relationship building and promoting recovery is a central premise in all interactions that nurses have with people and that nurses must have the knowledge and skills around communication to achieve a therapeutic alliance.

The study by Shinohara and colleagues used a randomised controlled trial methodology to investigate the effect of e‐learning on the topic of perinatal psychological support (2022). Participants included midwives and healthcare professionals (nurses and public health nurses working in perinatal care) and the outcome of interest was the development of empathic communication skills. Participants were randomised to receive an e‐learning education programme on perinatal psychological issues and empathic communication. The control group did not receive the education. Outcomes were assessed by a pre‐test, post‐test multiple choice questionnaire with a short case vignette, with responses used to evaluate the empathic communication skills. The second method of measurement was a video viewing of a simulated patient being interviewed by a midwife followed by the question ‘If you were in charge of the care of this woman, what would you say to her’? Verbatim responses were objectively assessed using the Empathic Understanding in Interpersonal Processes Scale (EUIP) (Carkhuff 1969 as cited in Shinohara et al. 2022). Scores across both methods of measurement moderately correlated (*r* = 0.409, *p* < 0.001) and were significantly higher in the intervention group (*p* < 0.0001), indicating that the education did increase empathic communication.

Opening space for communication or encouraging further expression are commonly used communication strategies found within these studies and they contribute substantially to the development of trust and rapport and enhanced safety for clinicians. Healthcare professionals that are educated in these techniques are more likely to apply an open and empathic approach to communication.

## 4 Discussion

This review highlights the centrality of communication to effective mental health practice, with converging evidence from observational, experimental, and qualitative research. Across diverse contexts and settings, communication strategies that emphasise empathy, presence, and validation were consistently linked to enhanced therapeutic engagement and recovery‐focused outcomes.

Opening space for communication or using an approach that encourages further dialogue are commonly used communication strategies found within the included studies and they contribute substantially to the development of trust and rapport. They position the client/patient at the centre of the exchange which promotes engagement and a real sense of authenticity. Studies consistently emphasised that patients must perceive healthcare professionals as genuinely engaged and attentive, with minimal distractions. This is particularly salient in mental health contexts, where the therapeutic relationship is often the primary intervention. The importance of non‐verbal communication—eye contact, posture, and tone—was also repeatedly highlighted, reinforcing the findings from Griep et al. (2016) regarding subtle, non‐verbal communicative behaviours that shape rapport. The careful use of non‐verbal communication reinforces this perception of presence (Molin and Graneheim 2022).

Trauma‐informed care (TIC) is a framework that supports the assumption that people who are seeking mental health care may have experienced trauma (Marks and Foster 2023). This assumption encourages health practitioners to focus on what has happened to the person, rather than the current issue. This provides the essential context of past experiences to enable understanding of the person's presenting behaviour and symptoms (Marks and Foster 2023). To optimise communication when working with people, it is imperative that all clinicians are cognisant of trauma‐informed care and how this concept shapes communication approaches. Trauma‐informed care within the mental health care context demands a more profound and systematic approach than what is required in the broader healthcare context.

Mindfulness and reflectivity as a practitioner are identified as essential professional qualities and are included in the professional Standards for Registered Nurses, Midwives and Social Workers (NMBA 2016; AASW 2023).

Dadirri is a concept and spiritual practice from the Aboriginal peoples of the Daly River region in the Northern Territory of Australia, particularly associated with the Ngangikurungkurr and Murrinhpatha peoples (Ungunmerr 2017). Dadirri is often translated as ‘deep listening’ of yourself through reflection and of others and whilst this communication technique has Indigenous origins it has a broader application (Ungunmerr 2017). Dadirri also prompts reflection and mindfulness alongside a connection to nature and country, all of which contributes to a therapeutic alliance and aligns with much of the evidence around attentive listening (Ungunmerr 2017; Finley et al. 2024; Berg et al. 2023; Chambers et al. 2015; Delaney et al. 2017). Potentially the ‘art’ of Dadirri and its associated techniques could be incorporated into professional practice. The use of silence, acknowledgement, and implicit empathy observed by Griep et al. reflects these broader professional strategies and demonstrates that even seemingly small communicative acts can enable deeper patient expression.

The recovery model is foundational to therapeutic approaches within the mental health paradigm. The Q‐methodology study by Lim et al. (2021) highlights nurses' conceptualisation of communication as inherently therapeutic and recovery‐oriented. The five enabling factors identified—acknowledgement, reassurance, exploration, collaboration, and encouragement—echo the sub‐themes of validation, individualised approaches, and strengths‐based care identified across the studies that used qualitative methodologies. Together, these studies suggest that communication is not merely an adjunct to practice but rather the basis of recovery‐oriented approaches. By explicitly recognising the emic experiences of people, nurses, midwives and social workers create opportunities to mitigate distress and reduce the likelihood of aggression, reinforcing the value of relational care in complex mental health contexts. Personal safety is an important consideration for healthcare professionals and enhanced safety for clinicians. Healthcare professionals who are educated in these techniques are more likely to apply an open and empathic approach to communication.

The randomised controlled trial by Shinohara et al. (2022) provides quantitative evidence that empathic communication can be actively taught and enhanced through structured education. The significant improvements in empathic communication scores among midwives and nurses who undertook e‐learning suggest that even brief interventions can have measurable impacts on professional practice. This finding resonates with the review's broader theme of professional mechanisms, particularly pre‐planning and reflective management of self, which underscore the value of deliberate preparation in communication.

Effective therapeutic communication within any context is multifaceted and this is particularly true within the mental health context. Evidence supports a range of strategies including reflective self‐management and mindfulness, intuitive attunement, humour, and planned therapeutic encounters. Importantly, the literature illustrates a dynamic tension between intuitive and planned communication: while experience‐derived intuition supports responsiveness and natural rapport, pre‐planning is essential in high‐risk contexts such as with restraint use or maternal–infant safety. Both approaches appear to serve complementary roles in practice, suggesting that flexible, context‐sensitive communication skills are necessary for optimal patient outcomes and that novice practitioners require the support of more experienced colleagues.

Collectively, these findings underscore that effective communication is both a skill and a stance: it involves teachable strategies, such as empathic phrasing and back‐channelling, but also requires an attitudinal commitment to presence, validation, and recovery‐focused care. The integration of observational, quantitative, and qualitative evidence suggests that communication training should not be considered an optional component of professional development but rather a central pillar of safe and effective practice. Future research should examine the long‐term sustainability of communication training, the interplay between intuitive and planned strategies, and the ways in which organisational contexts (such as staffing, environment, and culture) shape the capacity of healthcare professionals to enact therapeutic communication.

Communication with people within the mental health setting is fundamental to assessment and therapeutic approaches. The evidence to guide practice is somewhat limited but remains useful particularly for novice practitioners moving into the mental health space.

## 5 Limitations

It is essential to consider the quality of included studies and any methodological limitations when synthesising findings. The majority of studies included in this review were of moderate to good strength using the (Hong, Pluye, et al. 2018) tool.

The majority of the included studies focused on the discipline of nursing. We actively searched for more studies within midwifery and social work or studies with an interprofessional approach without great success. This will inform our recommendations for future research.

And finally, this scoping review was restricted to peer‐reviewed literature published in English, potentially excluding relevant studies in other languages and potentially introducing a language bias.

## 6 Implications for Future Research

The findings of this review highlight a significant gap in interprofessional research. Working within the contemporary mental health context demands an interprofessional approach and as such future research should incorporate relevant disciplines depending on the local context.

Additional gaps in the evidence include a paucity of research to support novice practitioners working within mental health. This is also true for students undertaking placement in a mental health setting. The future workforce will be better supported and integrated into the speciality by generating robust evidence to guide their practice.

## 7 Conclusion

The literature consistently highlights that effective communication is fundamental to quality mental health care. Strong communication skills enhance therapeutic relationships, promote shared decision‐making, and contribute to positive clinical outcomes. However, the evidence also reveals persistent challenges, including communication barriers arising from symptoms of mental illness, stigma, cultural misunderstandings, and interprofessional dynamics. Furthermore, novice practitioners, including social workers, midwives, and nurses, often report a lack of confidence in communication, underscoring the need for targeted training and support.

Despite these challenges, communication remains a modifiable factor with significant potential to improve patient engagement, safety, and recovery. Future research should explore the implementation of structured communication models in real‐world mental health settings and evaluate the impact of interprofessional education on collaborative communication. A focus on culturally responsive and trauma‐informed approaches will also be essential in addressing the needs of diverse populations.

## 8 Relevance for Clinical Practice

The findings of this review highlight that communication is a core skill essential to therapeutic effectiveness. Nurses, midwives, and social workers (particularly novices in the mental health setting) need to understand the evidence underpinning their practice as it demonstrates how purposeful, empathic, and recovery‐focused communication strategies foster trust, safety, and engagement. Techniques such as active listening, back‐channelling, and presence enhance rapport and relationship building. Embedding communication training and reflective practice within education and supervision can optimise therapeutic and recovery outcomes.

## Author Contributions

John Drayton: 50% concept; 33% writing and editing; 25% data extraction. Glenda Hawley: 25% concept; 33% writing and editing; 25% screening; 25% data extraction. Yee Kay Lai: 75% search strategy; 25% data extraction; 50% early drafts. Jacqueline Jauncey‐Cooke: 25% concept; 25% search strategy; 25% data extraction; 33% writing and editing; 25% screening.

## Funding

This review was supported by UQ funding for a Summer Scholarship to support Yee Kay to participate in this review.

## Conflicts of Interest

The authors declare no conflicts of interest.

## Data Availability

Data sharing not applicable to this article as no datasets were generated or analysed during the current study.

## References

[inm70371-bib-0001] Australian Association of Social Workers (AASW) . 2023. “AASW Practice Standards.” https://www.aasw.asn.au/about‐aasw/ethics‐standards/practice‐standards/.

[inm70371-bib-0002] Australian College of Mental Health Nurses . 2013. Scope of Practice of Mental Health Nurses in Australia 2013. ACMHN. https://www.acmhn.org/Web/Web/Resources/Best‐practice‐resources.aspx?hkey=c59b34ff‐f05a‐46a1‐9b27‐accc4288813b.

[inm70371-bib-0003] Australian Commission on Safety and Quality in Health Care . 2021. National Safety and Quality Health Service Standards. 2nd ed. ACSQHC. https://www.safetyandquality.gov.au/standards/nsqhs‐standards.

[inm70371-bib-0004] Berg, J. , E. Lipponen , E. Sailas , et al. 2023. “Nurses' Perceptions of Nurse–Patient Communication in Seclusion Rooms in Psychiatric Inpatient Care: A Focus Group Study.” Journal of Psychiatric and Mental Health Nursing 30: 781–794. 10.1111/jpm.12907. 36718606

[inm70371-bib-0005] Campbell, M. , J. E. McKenzie , A. Sowden , et al. 2020. “Synthesis Without Meta‐Analysis (SWiM) in Systematic Reviews: Reporting Guideline.” BMJ 368: l6890. 10.1136/bmj.l6890. PMC7190266 31948937

[inm70371-bib-0006] Chambers, M. , X. Kantaris , V. Guise , and M. Välimäki . 2015. “Managing and Caring for Service Users.” Journal of Psychiatric and Mental Health Nursing 22: 289–297. 10.1111/jpm.12199. 25944483

[inm70371-bib-0007] Clarivate . 2025. “EndNote (Version 2025.3).” https://endnote.com/.

[inm70371-bib-0008] Delaney, K. R. , M. Shattell , and M. E. Johnson . 2017. “Capturing the Interpersonal Process of Psychiatric Nurses: A Model for Engagement.” Archives of Psychiatric Nursing 31, no. 6: 634–640. 10.1016/j.apnu.2017.08.003. 29179832

[inm70371-bib-0009] Derblom, K. , J. Molin , S. Gabrielsson , and B. M. Lindgren . 2022. “Nursing Staff's Experiences of Caring for People With Mental Ill‐Health in General Emergency Departments: A Qualitative Descriptive Study.” Issues in Mental Health Nursing 43, no. 12: 1145–1154. 10.1080/01612840.2022.2138653. 36383445

[inm70371-bib-0010] Evans, E. C. , N. L. Deutsch , E. Drake , and L. Bullock . 2017. “Nurse–Patient Interaction as a Treatment for Antepartum Depression: A Mixed‐Methods Analysis.” Journal of the American Psychiatric Nurses Association 23, no. 5: 347–359. 10.1177/1078390317705449. 28459182

[inm70371-bib-0011] Finley, B. A. , K. D. Shea , S. P. Gallagher , and R. Taylor‐Piliae . 2024. “Psychiatric Mental Health Nurse Practitioners Experiencing Therapeutic Alliance While Using Tele‐Mental Health: A Phenomenological Study.” Archives of Psychiatric Nursing 49: 56–66. 10.1016/j.apnu.2024.01.016. 38734456

[inm70371-bib-0012] Griep, E. C. M. , J. Noordman , and S. van Dulmen . 2016. “Practice Nurses Mental Health Provide Space to Patients to Discuss Unpleasant Emotions.” Journal of Psychiatric and Mental Health Nursing 23, no. 2: 77–85. 10.1111/jpm.12279. 26710237

[inm70371-bib-0013] Hanan, K. , M. Peters , C. Godfrey , P. Mcinerney , and C. B. Soares . 2016. “An Evidence‐Based Approach to Scoping Reviews.” Worldviews on Evidence‐Based Nursing 13, no. 2: 118–123. 10.1111/wvn.12144. 26821833

[inm70371-bib-0014] Hong, Q. N. , S. Fàbregues , G. Bartlett , et al. 2018. “The Mixed Methods Appraisal Tool (MMAT) Version 2018 for Information Professionals and Researchers.” Education for Information 34, no. 4: 285–291. 10.3233/EFI-180221.

[inm70371-bib-0043] Hong, Q. N. , P. Pluye , S. Fàbregues , et al. 2018. “Mixed Methods Appraisal Tool (MMAT), Version 2018.” Registration of Copyright (#1148552). Canadian Intellectual Property Office, Canada.

[inm70371-bib-0015] International Confederation of Midwives (ICM) . 2024. Essential Competencies for Midwifery Practice. International Confederation of Midwives. https://internationalmidwives.org/resources/essential‐competencies‐for‐midwifery‐practice/.

[inm70371-bib-0016] International Council of Nurses (ICN) . 2020. “Position Statement, Revised Edition.” In Mental Health. Geneva: ICN. https://www.icn.ch/what‐we‐do/position‐statements.

[inm70371-bib-0017] Korteland, T. W. , K. M. Koorengevel , I. E. Poslawsky , and B. Meijel . 2019. “Nursing Interventions for Patients With Postpartum Psychosis Hospitalized in a Psychiatric Mother–Baby Unit: A Qualitative Study.” Journal of Psychiatric and Mental Health Nursing 26, no. 7–8: 254–264. 10.1111/jpm.12542. 31250503

[inm70371-bib-0018] Lim, E. , D. Wynaden , and K. Heslop . 2021. “Using Q‐Methodology to Explore Mental Health Nurses' Knowledge and Skills to Use Recovery‐Focused Care to Reduce Aggression in Acute Mental Health Settings.” International Journal of Mental Health Nursing 30, no. 2: 413–426. 10.1111/inm.12802. 33084220

[inm70371-bib-0019] Lin, Y.‐Y. , W.‐J. Yen , W.‐L. Hou , W.‐C. Liao , and M.‐L. Lin . 2022. “Mental Health Nurses' Tacit Knowledge of Strategies for Improving Medication Adherence for Schizophrenia: A Qualitative Study.” Health 10, no. 3: 492. 10.3390/healthcare10030492. PMC8955025 35326970

[inm70371-bib-0020] Marks, P. , and K. Foster . 2023. “Chapter 1—Introduction to Mental Health in Emergency Care.” In Mental Health in Emergency Care, 1–18. Elsevier ISBN: 9780729587976. 10.1016/B978-0-7295-4421-4.00010-9.

[inm70371-bib-0021] Molin, J. , and U. H. Graneheim . 2022. “Participation, Confirmation and Challenges: How Nursing Staff Experience the Daily Conversations Nursing Intervention in Psychiatric Inpatient Care.” Issues in Mental Health Nursing 43, no. 11: 1056–1063. 10.1080/01612840.2022.2116135. 36053790

[inm70371-bib-0022] Moxham, L. , M. Hazelton , E. Muir‐Cochrane , T. Heffernan , C. R. Kneisl , and E. Tigoboff . 2017. Contemporary Psychiatric‐Mental Health Nursing: Partnerships in Care/Lorna Moxham, Michael Hazelton, Eimear Muir‐Cochrane, Tim Heffernan, Carol Ren Kneisl, Eileen Tigoboff. Pearson Australia.

[inm70371-bib-0023] Noonan, M. , J. Jomeen , R. Galvin , and O. Doody . 2018. “Survey of Midwives' Perinatal Mental Health Knowledge, Confidence, Attitudes and Learning Needs.” Women and Birth: Journal of the Australian College of Midwives 31, no. 6: e358–e366. 10.1016/j.wombi.2018.02.002. 29454664

[inm70371-bib-0024] Nursing and Midwifery Board of Australia . 2016. “Registered Nurse Standards for Practice.” https://www.nursingmidwiferyboard.gov.au/Codes‐Guidelines‐Statements/Professional‐standards/registered‐nurse‐standards‐for‐practice.aspx.

[inm70371-bib-0025] Nursing and Midwifery Board of Australia . 2018. “Midwife Standards for Practice.” https://www.nursingmidwiferyboard.gov.au/Codes‐Guidelines‐Statements/Professional‐standards/Midwife‐standards‐for‐practice.aspx.

[inm70371-bib-0026] Oestergaard, C. U. , and B. Dinesen . 2019. “Video Communication as a Tool for Psychosocial Support for People Recovering From Severe Mental Disorder: Social Workers' Experiences.” mHealth 5: 38. 10.21037/mhealth.2019.08.09. PMC6789197 31620465

[inm70371-bib-0027] Open Science Framework (OSF) . 2025. “Registration of Review Title. Communication Within the Mental Health Setting: A Rapid Review.” https://osf.io/yftd8/.

[inm70371-bib-0028] Overpelt, N. , N. Kool‐Goudzwaard , B. Stringer , and B. Meijel . 2025. “Nurses' Perspectives on Fostering Therapeutic Relationships While Working With Clients Who Display Conflict Behaviour.” International Journal of Mental Health Nursing 34, no. 1: e13441. 10.1111/inm.13441. 39333839

[inm70371-bib-0029] Parakkal Kurian, L. , S. Rentala , C. X. Belsiyal , P. Srinivasan , and S. Govindar Thimmajja . 2023. “Aggressive Behavior and Its Associated Factors Among Persons With Mental Illness: An Observational Study.” Journal of Education Health Promotion 12: 249. 10.4103/jehp.jehp_195_23. PMC10506767 37727413

[inm70371-bib-0030] Saldaña, J. 2025. The Coding Manual for Qualitative Researchers. 5th ed. Sage Publishing.

[inm70371-bib-0031] Scheirich, L. , T. Maguire , and M. Daffern . 2024. “Testing a Recovery‐Oriented Nursing Communication Framework to Encourage Collaboration and Discussion About Aggression Prevention: A Mixed Methods Study.” International Journal of Mental Health Nursing 33, no. 5: 1303–1314. 10.1111/inm.13325. 38532536

[inm70371-bib-0032] Sharkiya, S. H. 2023. “Quality Communication Can Improve Patient‐Centred Health Outcomes Among Older Patients: A Rapid Review.” BMC Health Services Research 23: 886. 10.1186/s12913-023-09869-8. PMC10464255 37608376

[inm70371-bib-0033] Shinohara, E. , Y. Ohashi , A. Hada , and Y. Usui . 2022. “Effects of 1‐Day e‐Learning Education on Perinatal Psychological Support Skills Among Midwives and Perinatal Healthcare Workers in Japan: A Randomised Controlled Study.” BMC Psychology 10, no. 1: 133. 10.1186/s40359-022-00832-6. PMC9125975 35606868

[inm70371-bib-0034] Silverman, J. , S. Kurtz , and J. Draper . 2013. Skills for Communicating With Patients. 3rd ed. CRC Press. 10.1201/9781910227268.

[inm70371-bib-0035] Spielfogel, J. E. , and J. C. McMillen . 2017. “Current Use of de‐Escalation Strategies: Similarities and Differences in de‐Escalation Across Professions.” Social Work in Mental Health 15, no. 3: 232–248. 10.1080/15332985.2016.1212774.

[inm70371-bib-0036] Stevens, A. , M. Hersi , C. Garritty , et al. 2025. “Rapid Review Method Series: Interim Guidance for the Reporting of Rapid Reviews.” BMJ Evidence‐Based Medicine 30: 118–123. 10.1136/bmjebm-2024-112899 PMC12013547 39038926

[inm70371-bib-0037] Tolosa‐Merlos, D. , A. R. Moreno‐Poyato , F. González‐Palau , A. Pérez‐Toribio , G. Casanova‐Garrigós , and P. Delgado‐Hito . 2023. “Exploring the Therapeutic Relationship Through the Reflective Practice of Nurses in Acute Mental Health Units: A Qualitative Study.” Journal of Clinical Nursing 32, no. 1–2: 253–263. 10.1111/jocn.16223. PMC10078778 35075705

[inm70371-bib-0038] Ungunmerr, M.‐R. 2017. “To Be Listened to in Her Teaching: Dadirri: Inner Deep Listening and Quiet Still Awareness.” EarthSong Journal: Perspectives in Ecology, Spirituality and Education 3, no. 4: 14–15. 10.3316/informit.732386012034745.

[inm70371-bib-0039] Unhjem, J. V. , S. Vatne , and M. H. Hem . 2018. “Transforming Nurse–Patient Relationships—A Qualitative Study of Nurse Self‐Disclosure in Mental Health Care.” Journal of Clinical Nursing 27, no. 5–6: e798–e807. 10.1111/jocn.14191. 29193417

[inm70371-bib-0040] Vandewalle, J. , D. Beeckman , A. Van Hecke , B. Debyser , E. Deproost , and S. Verhaeghe . 2019. “Contact and Communication With Patients Experiencing Suicidal Ideation: A Qualitative Study of Nurses' Perspectives.” Journal of Advanced Nursing 75, no. 11: 2867–2877. 10.1111/jan.14113. 31222813

[inm70371-bib-0041] Xue, W. , and C. Heffernan . 2021. “Therapeutic Communication Within the Nurse–Patient Relationship: A Concept Analysis.” International Journal of Nursing Practice 27, no. 6: e12938. 10.1111/ijn.12938. 33949044

[inm70371-bib-0042] Zimmermann, C. , L. Del Piccolo , J. Bensing , et al. 2011. “Coding Patient Cues and Concerns in Medical Consultations: The Verona Coding Definitions of Emotional Sequences (VR‐CoDES).” Patient Education and Counseling 82: 141–148. 10.1016/j.pec.2010.03.017 20430562

